# Fibromatous Uterus in a 16-Year-Old Girl: A Case Report

**DOI:** 10.1155/2010/932762

**Published:** 2010-11-28

**Authors:** Athina C. Tsili, Ekaterini D. Lentoudi, Maria I. Argyropoulou, Nikolaos Dalkalitsis, Anna Batistatou, Evangelos Paraskevaidis, Konstantine Tsampoulas

**Affiliations:** ^1^Department of Clinical Radiology, University Hospital of Ioannina, 45110 Ioannina, Greece; ^2^Department of Obstetrics & Gynaecology, University Hospital of Ioannina, 45110 Ioannina, Greece; ^3^Department of Pathology, University Hospital of Ioannina, 45110 Ioannina, Greece

## Abstract

Although uterine leiomyomas are the most common neoplasms of the female genital tract, this is not the case when referring to women under the age of 20. Only a few cases of uterus leiomyomas have been reported in this age. Preoperative imaging evaluation is mandatory in adolescent women for the accurate detection, localization, and characterization of uterus leiomyomas. We report a case of a 16-year-old girl admitted to our hospital for pain and abdominal distention. The patient underwent multidetector CT examination of the abdomen and MR examination of the pelvis. Both imaging modalities revealed uterine enlargement and the presence of innumerable variably sized leiomyomas. Histopathologic examination following exploratory laparotomy confirmed the presence of uterus leiomyomas. The patient underwent laparoscopic myomectomy two years after the first operation, following MR examination of the pelvis.

## 1. Introduction

Leiomyomas, also known as fibroids or fibromas represent the most common uterine neoplasm, occurring in 20–30% of women between the ages of 35 and 50 [1–4]. However, these benign tumors are extremely rare in women under the age of 20 [5–11]. An accurate detection, characterization and localization of uterine leiomyomas are important in these patients. MR imaging is considered the examination of choice for the detection and localization of uterus fibroids [1–4, 12, 13]. Uterine leiomyomas represent an incidental finding on CT examination, usually performed for a variety of other reasons [4, 14]. 

We present a case of a 16-year-old girl with fibromatous uterus, evaluated with multidetector CT and MR imaging examination. As to our knowledge, this is the first report of a uterus with multiple fibroids in an adolescent girl in the English literature, although there are few reports of solitary uterus leiomyomas in this age population [5–11]. The value of preoperative imaging evaluation in these patients is discussed.

## 2. Case Report

A 16-year-old female patient was referred to the emergency unit of our hospital for abdominal pain and distention. Patient's gynecologic history was unremarkable. Menarche occurred at the age of 13, and menses had been regular ever since. From the family history, her mother reported diabetes mellitus.

Physical examination revealed the presence of a relatively firm pelvic mass, of probably uterine origin. Laboratory analysis showed a mild anemia and a slight elevation of CA 125 levels (40 U/ml). The possibility of pregnancy was excluded after a negative pregnancy test.

**Figure 1:**
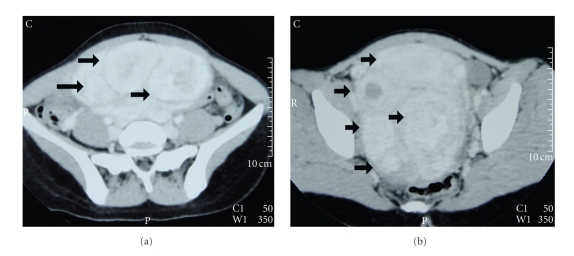
Transverse postcontrast CT images (portal phase) reveal numerous uterine leiomyomas of variable size (arrows). The masses show inhomogeneous enhancement after contrast material administration.

Ultrasonography, both transabdominal and transvaginal, showed globular uterus enlargement and multiple hypoechoic or heterogeneous masses, probably representing leiomyomatous cores, causing distortion of the central endometrial echo. Multidetector CT examination of the abdomen on a 16-row CT scanner was followed. On CT, an enlarged uterus, with lobular, deformed contour was detected, filling the pelvis and extending up to the level of the lower pole of the kidneys. Multiple uterus leiomyomas, of variable size were found, heterogeneously enhancing after contrast material administration (Figure 1). Neither ascites, nor lymphadenopathy was revealed. No renal hydronephrosis was noted.

**Figure 2:**
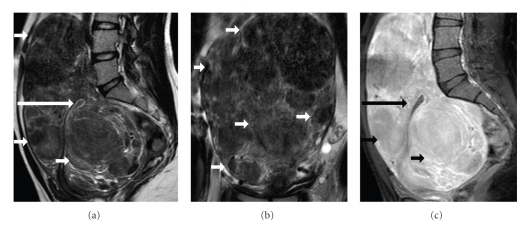
Sagittal (a) and coronal (b) T2-weighted MR images show uterus enlargement. Multiple, relatively homogeneous fibroids are revealed (arrows), of low signal intensity, causing significant compression of the uterine endometrial cavity (long arrow). (c) Sagittal fat-suppressed T1-weighed image depicts strong and heterogeneous lesion enhancement (arrows).

Finally, the patient underwent MR imaging examination of the pelvis on a 1.5 Tesla unit. An enlarged, posteriorly inclined uterus was found (Figure 2). The presence of innumerable intramural uterus leiomyomas was confirmed, of maximal diameter 13 cm, detected mainly of low signal intensity on T2-weighted images, when compared to the outer myometrium (Figure 2(a) and 2(b)), slightly inhomogeneous the larger ones. The masses were well circumscribed, isointense to the adjacent myometrium on T1-weighted images, with contrast enhancement (Figure 2(c)). Both ovaries were normal. 

**Figure 3:**
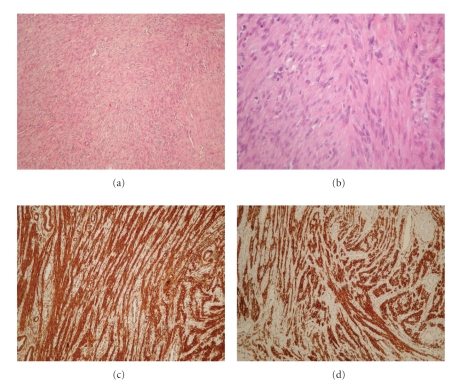
Microscopic features of the largest nodule (of 13 cm in diameter). (a) Spindle cells arranged in interlacing bundles, within a collagenous stroma (H+E, ×200). (b) The cells have vesicular relatively uniform nuclei (H+E, ×400). (c) Leiomyomatous cells express SMA (DAB ×200). (d) Leiomyomatous cells express desmin (DAB ×200).

Imaging findings were diagnostic for the presence of fibromatous uterus. The patient underwent exploratory laparotomy. An extremely enlarged uterus, with multiple and variably sized fibroids, the largest of which about 10 cm in maximal diameter was found in surgery. Frozen section pathologic examination confirmed the presence of uterus leiomyomas. Most of leiomyomas were excised; some left in place due to their close relation to the uterine vessels. For histologic examination, 19 discrete fairly well-circumscribed nodules were received. They measured 1–13 cm in maximal diameter. On cut section, the nodules were well whitish, with whorled appearance and fibroelastic consistency. No areas of hemorrhage or necrosis were found. On microscopic examination, all nodules were composed of relatively uniform spindle cells with vesicular nuclei, arranged mostly in interlacing bundles and embedded within a collagenous stroma (Figure 3(a) and 3(b)). The mitoses were rare (max. 1 mitosis/10 high power fields). Immunohistochemical examination showed cell positivity for smooth muscle actin (SMA, Figure 3(c)) and desmin (Figure 3(d)). Based on the above, the diagnosis of multiple leiomyomas was made.

**Figure 4:**
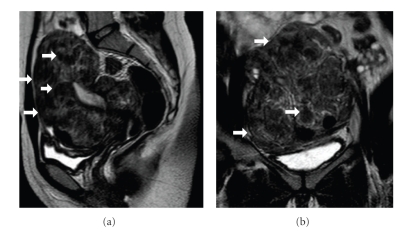
Followup MR imaging study—two years after surgery. Sagittal (a) and coronal (b) T2-weighted images reveal decrease of the uterine size. Multiple leiomyomas of low to intermediate signal intensity (arrows) are detected.

The patient was instructed to have pelvic follow-up sonograms on 6-months intervals. MR imaging examination performed two years after surgery, when the patient was admitted to the Gynecology clinic with lower abdominal pain, revealed recurrence of fibromatous uterus (Figure 4). Laparoscopic myomectomy was followed. Five well-circumscribed nodules measuring 0.7–1.3 cm in maximal diameter were histologically examined. The macroscopic and microscopic features were identical to those in the previous specimen and the diagnosis of uterus leiomyomas was confirmed.

## 3. Discussion

Uterus leiomyomas are extremely uncommon in the paediatric and adolescent population [5–11]. Approximately twelve cases of uterine leiomyomas among teenagers, under the age of 18 years have been reported in the English language literature [5–11]. In all these cases, a solitary symptomatic, large uterus fibroid was described. Our case is one of the first reporting a uterus with multiple fibroids in an adolescent girl.

Estrogens and progesterone play an important role in the development of these neoplasms [1, 5–8, 15]. Uterus leiomyomas are estrogen-dependent tumors and exogeneous estrogen, obesity and pregnancy usually influence their growth [1, 5–8, 15]. Pregnancy and obesity were excluded in our patient as well as any history of administration of pharmaceutical agents. A genetic component in the pathogenesis of uterine leiomyomas has also been strongly suggested [16, 17]. Inheritance may play an important role, as indicated by family and twin-pair studies, although no positive family history was reported in this patient. Cytogenetic abnormalities involving chromosomes 6, 7, 12, and 14 have been reported in uterus fibroids with high frequency, although relevant studies were not performed in this case [16, 17].

Leiomyomas in the young population often show histologic features favouring the diagnosis of malignancy; half of the reported cases demonstrated increased cellularity, mitotic activity, and cellular atypia [5]. These pathologic characteristics were not met in our patient. 

Uterine leiomyomas, although rare they should be considered in adolescent women presenting with a pelvic mass and abdominal pain, as in this case, or menstrual disorders and abnormal uterine bleeding. The management of leiomyomas in this age should be conservative for the preservation of fertility. Therefore, the preoperative characterization of the nature of these tumors is extremely important. The diagnosis should be based on imaging findings, that is, sonographic and magnetic resonance imaging features.

Ultrasonography is well established as the primary method for the evaluation of the female genital tract [4, 18]. It is a noninvasive, widely available technique, with satisfactory results in the detection of uterus fibroids [4, 18]. Although, CT is not recommended for the evaluation of uterine leiomyomas, radiologists should be familiar with the CT findings of these benign neoplasms, since they are often found, either incidentally on a CT examination performed for a variety of other reasons, or for the investigation of abdominal pain [4, 14]. The typical CT findings of leiomyomas include an enlarged uterus, with a lobular, deformed contour. Leiomyomas usually manifest as homogenous masses, similar to the normal myometrium, or as heterogeneously enhancing lesions. The above findings were seen in our patient.

MR imaging is considered the most accurate technique for the detection and localization of leiomyomas, proved more accurate than US [1, 2, 4, 12, 13, 19–22]. MRI can assist in preoperative planning for myomectomy by accurately depicting and localizing uterine leiomyomas. The technique has proved superior to sonography in localization of leiomyomas, especially in cases of enlarged, myomatous uterus, as it was in this patient. Differential diagnosis from conditions that may mimic uterine leiomyoma both clinically and sonographically, such as adenomyosis, adnexal tumor, or focal myometrial contraction, is possible with MR imaging. Adnexal masses are more common in women under the age of 20, therefore differential diagnosis from uterus leiomyomas is particularly important in this age population [9]. The superb contrast, multiplanar capability combined with the absence of ionizing radiation render MR imaging the modality of choice in detecting and characterizing tumors. Typical findings of uterine leiomyomas at MR examination are well known, including sharply demarcated uterine masses, homogeneously hypointense compared to the normal myometrium on T2-weighted images [1]; therefore diagnosis is usually straightforward, as proven also in our case.

Differential diagnosis of uterus leiomyomas should also include uterus leiomyosarcoma [1]. Although, the presence of a rapidly growing or an irregularly marginated uterus leiomyoma has been proposed as suggestive of malignant transformation, the final diagnosis of uterus leiomyosarcoma is established mainly on histopathologic findings [1].

## Abbreviations

CT: Computed tomography
MRI: Magnetic resonance imaging
CA: Cancer antigen
SMA: Smooth muscle actin
US: Ultrasonography.

## References

[1] Murase E, Siegelman ES, Outwater EK, Perez-Jaffe LA, Tureck RW (1999). Uterine leiomyomas: histopathologic features, MR imaging findings, differential diagnosis, and treatment. *Radiographics*.

[2] Hricak H, Tscholakoff D, Heinrichs L (1986). Uterine leiomyomas: correlation of MR, histopathologic findings, and symptoms. *Radiology*.

[3] Cornfeld D, Israel G, Martel M, Weinreb J, Schwartz P, McCarthy S (2010). MRI appearance of mesenchymal tumors of the uterus. *European Journal of Radiology*.

[4] Karasick S, Lev-Toaff AS, Toaff ME (1992). Imaging of uterine leiomyomas. *American Journal of Roentgenology*.

[5] Grapsa D, Smyrniotis V, Hasiakos D, Kontogianni-Katsarou K, Kondi-Pafiti A (2006). A giant uterine leiomyoma simulating an ovarian mass in a 16-year-old girl: a case report and review of the literature. *European Journal of Gynaecological Oncology*.

[6] Bekker G, Gavrilescu T, Rickets-Holcomb L, Puka-Khandam P, Akhtar A, Ansari A (2004). Symptomatic fibroid uterus in a 15-year-old girl. *International Surgery*.

[7] Diesen DL, Price TM, Skinner MA (2008). Uterine leiomyoma in a 14-year-old girl. *European Journal of Pediatric Surgery*.

[8] Fields KR, Neinstein LS (1997). Uterine myomas in adolescents: case reports and a review of the literature. *Journal of Pediatric and Adolescent Gynecology*.

[9] Perkins JD, Hines RS, Prior DS (2009). Uterine leiomyoma in an adolescent female. *Journal of the National Medical Association*.

[10] Michala L, Vlachos GD, Belitsos P, Antsaklis A (2010). Uterine fibroid in an adolescent: an unlikely diagnosis?. *Journal of Obstetrics and Gynaecology*.

[11] Berveiller P, Mir O, Menu Y, Jamali M, Carbonne B (2010). Fertility-sparing approach in a teenager with uterine tumor diagnosed as a sarcoma on imaging. *Gynecologic and Obstetric Investigation*.

[12] Hamlin DJ, Pettersson H, Fitzsimmons J, Morgan LS (1985). MR imaging of uterine Leiomyomas and their complications. *Journal of Computer Assisted Tomography*.

[13] Okizuka H, Sugimura K, Takemori M, Obayashi C, Kitao M, Ishida T (1993). MR detection of degenerating uterine leiomyomas. *Journal of Computer Assisted Tomography*.

[14] Casillas J, Joseph RC, Guerra JJ (1990). CT appearance of uterine leiomyomas. *Radiographics*.

[15] Rein MS, Barbieri RL, Friedman AJ (1995). Progesterone: a critical role in the pathogenesis of uterine myomas. *American Journal of Obstetrics and Gynecology*.

[16] Ligon AH, Morton CC (2001). Leiomyomata: heritability and cytogenetic studies. *Human Reproduction Update*.

[17] Ligon AH, Morton CC (2000). Genetics of uterine leiomyomata. *Genes Chromosomes and Cancer*.

[18] Freimanis MG, Jones AF (1992). Transvaginal ultrasonography. *Radiologic Clinics of North America*.

[19] Zawin M, McCarthy S, Scoutt LM, Comite F (1990). High-field MRI and US evaluation of the pelvis in women with leiomyomas. *Magnetic Resonance Imaging*.

[20] Mayer DP, Shipilov V (1995). Ultrasonography and magnetic resonance imaging of uterine fibroids. *Obstetrics and Gynecology Clinics of North America*.

[21] Riccio TJ, Adams HG, Munzing DE, Mattrey RF (1990). Magnetic resonance imaging as an adjunct to sonography in the evaluation of the female pelvis. *Magnetic Resonance Imaging*.

[22] Weinreb JC, Barkoff ND, Megibow A, Demopoulos R (1990). The value of MR imaging in distinguishing leiomyomas from other solid pelvic masses when sonography is indeterminate. *American Journal of Roentgenology*.

